# COVID 19—impact on substance use treatment utilization and provision in South Africa

**DOI:** 10.1186/s13011-022-00446-6

**Published:** 2022-03-03

**Authors:** Nadine Harker, Kim Johnson, Jodilee Erasmus, Bronwyn Myers

**Affiliations:** 1 Alcohol, Tobacco and Other Drug Research Unit, South African Medical Research Council, 1 Francie van Zyl Drive, Parow Valley, Cape Town, South Africa; 2 School of Public Health and Family Medicine, University of Cape Town, Cape Town, South Africa; 3 Curtin enAble Institute, Faculty of Health Sciences, Curtin University, Perth, WA Australia; 4 Division of Addiction Psychiatry, Department of Psychiatry and Mental Health, University of Cape Town, Cape Town, South Africa

**Keywords:** COVID-19, Treatment utilisation, South Africa, Substance use disorders

## Abstract

**Background:**

The coronavirus disease 2019 (COVID-19) pandemic has impacted people with substance use disorders (SUDs) worldwide. The aim of this study was to explore, changes in the number of SUD treatment episodes provided during the height of the pandemic and, SUD treatment providers’ perceptions of the impact of COVID-19-related restrictions on people with SUDs and the delivery of SUD treatment services in South Africa.

**Methods:**

We used administrative data collected as part of the South African Community Epidemiology Network on Drug Use (SACENDU) project to assess whether the number of treatment episodes changed during the height of COVID-19 restrictions. We used data from an online survey of SUD treatment providers to assess providers’ perceptions of the impact of COVID-19 on SUD treatment delivery. Eight seven SUD facilities were recruited to participate in the online survey.

**Results:**

Sixty-three organisations (out of a total of 86) participated in the survey, yielding a 73.2% response rate. About half (*n* = 30; 47.6%) of the sample thought the need for SUD treatment had remained the same or had increased during the COVID-19 lockdown. Half the sample (*n* = 32; 50.7%) reported decreased availability of SUD services during COVID-19 lockdowns. Participants believed that the lack of services during COVID-19 lockdown impacted negatively on patients that were enrolled in their programmes and on individuals who wished to access the service. Furthermore, changes in service provision seemed to increase patients’ anxiety, exacerbate pre-existing mental health problems and in some cases were thought to precipitate relapse. In addition, patient disengagement and attrition from treatment were thought to have increased during this period. Whilst 47.6% (*n* = 30) of providers agreed with the value of the alcohol ban, 23.8% (*n* = 15) of providers thought it had unintended negative consequences.

**Conclusion:**

Based on the findings it is evident that SUD treatment services in South Africa have been significantly affected during the COVID-19 pandemic and more severely during the onset of the pandemic. Together with service providers, more effective ways should be sought on how to feasibly expand access to SUD treatment for all South Africans and enhance the country’s preparedness for future health emergencies.

## Background

In 2020, the World Health Organisation (WHO) declared the novel 2019-nCoV (coronavirus disease 2019) a global pandemic [1–3]. In response, countries have taken drastic actions to curb the rate of COVID-19 infections including implementing rapid mass testing of the population and physical distancing measures such as strict community and country lockdowns [4]. In South Africa, the National Department of Health established its advisory council on coronavirus management team, modelled on the WHO’s Framework for a Public Health Emergency Operations Centre and implemented a national country lockdown to slow down and stop the spread of the virus, first implemented on 26 March 2020 [5]. During this “level 5” lockdown, people’s mobility was limited to leaving home for the acquisition of essential items such as food and medical supplies, with all non-essential services being halted. This lockdown included the complete prohibition of the sale and distribution of alcohol and tobacco. The decision to ban alcohol and tobacco sales was aimed at lowering alcohol-related emergency care visits and hospitalizations and tobacco-related risk of severe COVID-19 disease in the hope of freeing up health system capacity for managing the growing number of COVID-19 cases in the country [6]. Since the country’s initial and highly restrictive lockdown, there has been an intermittent lifting and reinstating of lockdown measures (including alcohol restrictions) in response to changing COVID-19 community transmission rates.

In a context where COVID-19 vaccines were not available to the general population until April 2021, these lockdown measures were necessary for controlling the rate of new infections and protecting health system capacity. However, as these measures also restricted the type of services that substance use disorder (SUD) services could provide, there may have been unintended consequences on people who use substances.

The impact of the COVID-19 pandemic and related restrictions on people with SUDs is of global concern [1]. Some studies have demonstrated increased use of alcohol and other substances among people with SUDs to cope with pandemic-related stress and uncertainty. In addition, changes in drug market supply arising from border closures, tighter alcohol regulations, and movement restrictions may lead to people with SUDs engaging in patterns of substance use associated with greater risk of harm [7]. For instance, in the absence of their substance of choice, people may switch to a more potent substance (for instance, from alcohol to other drugs) or change their preferred route of administration (such as from smoking to injection drug use) to avoid symptoms of withdrawal [8]. Many people who are unable to secure a supply of drugs may experience acute withdrawal symptoms, requiring specialist SUD treatment [4]. Despite the greater risk of harms, emerging evidence suggests that access to SUD treatment has become severely curtailed across the world [1]. This is partly because SUD services in many countries were considered non-essential during the pandemic which allowed for health resources to be directed away from SUD services towards treating and managing COVID-19 [1, 9].

Little is known about the impact of COVID-19 restrictions on access to and use of SUD services in South Africa. South Africa’s SUD treatment system comprises a mix of residential and outpatient (ambulatory) services, provided largely by private, not-for-profit organisations who receive state-funding [10]. The bulk of the estimated 20,000 treatment episodes that take place annually in South Africa occur through community-based outpatient services [11]. The sudden introduction of restrictive lockdown measures is likely to have impacted on these organisations’ ability to continue to provide services, but the extent to which SUD treatment provision has been compromised and the impact of these restrictions on service users during a time of restricted access to alcohol and other substances has not been documented. This information is needed to guide efforts to protect the South African SUD treatment system against the effects of future pandemics and health emergencies.

In response to this need, this study explored (i) changes in the number of SUD treatment episodes provided during the height of the COVID-19 pandemic and (ii) SUD treatment providers’ perceptions of the impact of COVID-19-related restrictions on people with SUDs and the delivery of SUD treatment services in South Africa.

## Methods

We used administrative data collected as part of the South African Community Epidemiology Network on Drug Use (SACENDU) project [11] to assess whether the number of treatment episodes changed during the height of COVID-19 restrictions. We used data from an online survey of SUD treatment providers to assess providers’ perceptions of the impact of COVID-19 on SUD treatment delivery.

Since 1996, the SACENDU project has collected data on all patients admitted to specialist SUD services across nine provincial sites on a six-monthly basis [11]. There are currently 87 (102 with the inclusion of satellite offices) SUD facilities participating in the SACENDU network, representing about 80% of all providers in the country. Treatment providers at participating facilities complete a SACENDU treatment admission form that includes questions on the demographic characteristics, patterns of substance use and treatment history of each patient enrolled into services. Data from each treatment centre are aggregated to allow for provincial and regional trends on the number of SUD treatment episodes provided during the six-month period and changes in the demographic and clinical characteristics of people entering treatment. In this paper, we will explore changes in the number of SUD treatment episodes provided between January 2017 and December 2020.

For the online survey of SUD treatment providers, we invited key personnel (directors, programme managers, social workers and administrators) from treatment facilities participating in SACENDU to complete a brief online survey of the impact of COVID-19 on their operations. Potential participants were contacted via telephone or email and asked to participate in the online survey. A follow up email containing the link to the survey was emailed to all individuals who agreed to participate. Providers were requested to complete the survey within two weeks. Participants were required to complete an online informed consent form prior to beginning the survey. We provided telephonic and email reminders to providers who did not complete the survey within the requested time.

The questionnaire contained 16 forced-choice and open-ended questions that explored characteristics of the treatment facility and types of services provided, perceived changes in access to SUD treatment and harm reduction services, challenges to providing SUD treatment during COVID-19 lockdowns, and perceived impact of COVID-19 on people utilising their treatment service. Stellenbosch University’s Faculty of Health Science’s Human Research Ethics Committee provided ethical approval for this study (HREC number N10-08–253).

### Data analysis

For the close-ended questions, statistics were computed using the Statistical Package for the Social Sciences (Norusis/SPSS Inc., 1988). Descriptive analyses such as basic frequency tabulations were conducted to describe the demographic profile of patient centres, the types of services offered, access to specialist care and harm reduction services, availability of substances, challenges faced by organisations tasked with rendering specialist care, as well as perception on the potential implications of the restrictions that were imposed on the sale of alcohol and tobacco. For the open-ended question, two of the authors coded the responses, meeting regularly to discuss codes, and resolve any discrepancies until they came up with a final list of codes.

For the SACENDU data, descriptive analyses were conducted to describe treatment admissions for substance use (alcohol included) during the 2017–2020 period.

## Results

### Profile of participating organisations

Sixty-three organisations (out of a total of 87), ranging in intensity of treatment provided (outpatient vs residential) participated in the survey, yielding a 72.4% response rate. Non-profit organisations formed the bulk of participating facilities (57.1%). Most (61.9%) of the organisations were residential facilities. Participating facilities were located primarily in the Western Cape and Gauteng Province, the two provinces with the largest concentration of SUD facilities [10]. Facilities usually treated a median of 30 patients a month (interquartile range 5–200). Table 1 presents the organisational characteristics of the sample, and that of the larger population of facilities participating in the SACENDU network.

**Table 1:** Profile of organisation participating in the survey (*n* = 63) and in the SACENDU network (*n* = 86) during the same time period (2020)

**Source of funding**	**N (%) of responses to the online survey (*n* = 63)**^b^	**N among facilities in the SACENDU network**^a^ **(*n* = 87**
Non-profit organisation	36 (57.1%)	60 (69%)
Private	18 (28.5%)	18 (20.7%)
State-owned facility	9 (14.2%)	9 (10.3%)
**Intensity of treatment provided**^b^		
Residential	39 (61.9%)	53^c^(60.9%)
Outpatient	24(38.0%)	34 (39.1%)
**Provincial distribution** *(number of centres participating)*		
Eastern Cape	4 (6.3%)	7 (8%)
Free State	2 (3.1%)	3 (3.4%)
Gauteng	13 (20.6%)	26 (29.9%)
Kwazulu- Natal	6 (9.5%)	14 (16.1%)
Limpopo	2 (3.1%)	5 (5.7%)
Mpumalanga	7 (11.1%)	7 (8%)
Northern Cape	1 (1.5%)	1 (1.1%)
Western Cape	28 (44.4%)^d^	23 (26.4%)
North West	0	1 (1.1%)

^a^*N* = exclusion of satellite offices within provinces

^b^1 centre unidentified

^c^includes 15 dual residential/outpatient centres

^d^included satellite offices

### Need for and availability of SUD treatment services during COVID-19

About half (*n* = 30; 47.6%) of the sample thought the need for SUD treatment had remained the same or had increased during the COVID-19 lockdown, based on the experiences of treatment enquiries. Yet half the sample (*n* = 32; 50.7%) reported decreased availability of SUD services during COVID-19 lockdowns to meet this demand for SUD services.

They attributed changes in the availability of services to physical distancing regulations and movement restrictions implemented during the country’s national lockdown. Of the 63 participating organisations, only a third (*n* = 20, 31.7%) reported that they had remained fully operational during the most restrictive lockdowns. The remaining 43 organisations either closed (*n* = 7; 11.0%) or limited the range of services they provided (*n* = 36; 57.1%). Among the 36 facilities that adjusted the way they provided services, the majority (*n* = 21, 58%) reported switching from face-to-face to virtual service delivery via telephone or videoconferencing as a means of facilitating continued care. Others (*n* = 5, 13%) reported limiting services to existing clients but pausing new enrolments or only providing a referral service (*n* = 10, 27%), referring clients who needed immediate care to other organisations who were still operating.

**Fig. 1:**
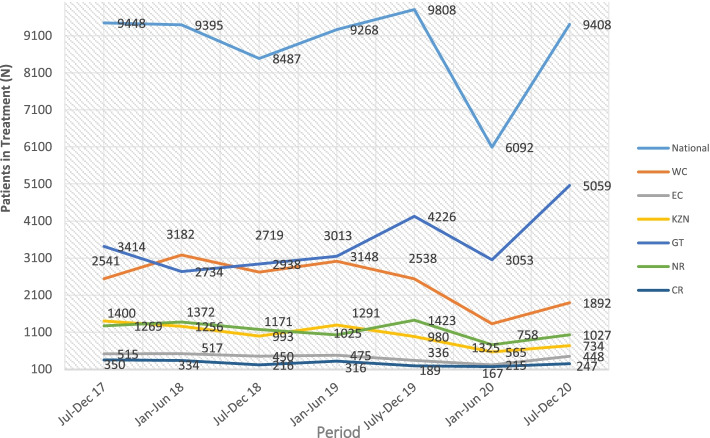
Patients in treatment – SACENDU data (2017–2020)

This corresponds with data from the SACENDU network that indicates a decrease in the utilization of SUD treatment during the national lockdown. The SACENDU data indicates a decrease in the number of treatment admissions in the first half of 2020 (corresponding to the most restrictive period of national lockdown) when compared to 2019 and preceding years (Fig. 1). During the second half of 2020 as lockdown eased, the number of treatment admissions began to increase but remained below the numbers seen in previous years. This trend was observed in all the provinces (see Fig. 1).

### Perceived impact on patients of COVID-19 changes to SUD service delivery

In response to open-ended questions about how changes to SUD services affected patients, most participants believed that the lack (or limited availability) of services during COVID-19 lockdown impacted negatively on patients that were enrolled in their programmes and on individuals who wished to access the service (potential new enrolments). During the level 5 lockdown period, the few facilities that provided pharmacotherapy for opioid use disorder as part of their programme were closed. Service providers reported that reductions in the amount and kind of support provided to patients during this time seemed to increase patients’ anxiety, exacerbate pre-existing mental health problems and in some cases were thought to precipitate relapse to substance use after a period of abstinence or migrating to other substances to deal with their cravings. Participants noted that these changes also impacted on the broader family structure who now had to take on additional support responsibilities but often lacked the skills and knowledge of how best to support people struggling with substances.

In addition, service providers noted that rates of patient disengagement and attrition from treatment increased during this period. Even though some services were adapted to be COVID-19 compliant, many patients did not utilise the treatment options available due to lack of mobile technology to support the use of virtual platforms, data issues or privacy concerns. Provider reports suggest that this was due to a variety of financial and practical barriers to care engagement. For those offering face-to-face sessions, providers noted that transportation costs and restrictions on movement (without adequate guidelines on how to obtain permissions to access essential care treatment) were a barrier to patient participation. Additionally, the broader family who would have ordinarily provided some financial assistance was unable to because of economic hardship sustained during COVID. Providers were also of the opinion that COVID-related fears and loss of confidence in the quality of treatment (related to COVID-19 related changes to the range of services provided and a narrowing of the treatment offering) further impacted on patients’ engagement in treatment. Attempts were made to contact clients telephonically to initiate contact and provide telephonic therapeutic support but mostly with no success. Providers reported that patients rarely answered their phones, which they thought was due to limited access to a mobile phone, limited telephone coverage and privacy concerns.

### Providers’ perceptions of the impact of the alcohol and tobacco bans on patients enrolled within SUD services.

Most providers thought that the availability of alcohol and tobacco had decreased during the COVID-19 lockdowns but reported not knowing whether there had been any changes in the availability of other drugs.

Whilst 47.6% (*n* = 30) of providers agreed with the value of the alcohol ban, 23.8% (*n* = 15) of providers thought it had unintended negative consequences. Among these participants, the most representatively held view was that it encouraged the illegal purchase of alcohol (73%, *n* = 46), followed by the view that it led to the home-brewing of alcohol which may have had more adverse health consequences than formally produced alcohol (54%, *n* = 34). Participants also expressed concern that the limited access to alcohol (58.7%, *n* = 37) or tobacco (82.0%, *n* = 52) may have increased feelings of anxiety and distress among patients receiving treatment at the time of the ban.

### Patient and provider access to COVID-19 guidelines, information and personal protective equipment

All centres reported some form of communication with their patients on COVID-related guidelines and safety measures, with 82% indicating communication that included sharing of information in-person, through mobile text messaging and virtual messaging platforms (WhatsApp), as well as print materials (posters and pamphlets). Only one organisation reported no communication about COVID-safety protocols with patients. With regard to guidelines for the prevention and management of COVID-19 that are tailored for the provision of substance use treatment services, only 65.0% (*n* = 41) of organisations were aware that some guidelines existed. Most providers (76.2%, *n* = 48) indicated that staff had received some training in how to manage COVID-19 risks, but 23.0% (*n* = 15) reported that no training had taken place by the time of the survey. There were also varied responses about staff access to personal protective equipment (PPE) with 74.6% (*n* = 47) of providers noting that their organisations had sufficient access, 11.1% (*n* = 7) reporting variable access to PPE at their organisation, and 14.3% (*n* = 9) reporting that their organisation had not provided PPE to staff.

## Discussion

Globally, the coronavirus pandemic presented unique challenges for individuals who use substances as well as the provision of treatment for SUDs [1, 9]. However, the impact of COVID-19 lockdown measures on SUD treatment in South Africa, a country that imposed among the most stringent lockdown restrictions globally, is not well understood. In this study we explored SUD providers perceptions of how South Africa’s responses to the pandemic impacted on individuals seeking SUD treatment and the delivery of SUD treatment services in South Africa.

Most importantly, and in keeping with studies from other contexts [9], our findings highlight diminished access to SUD treatment during this period. Although providers thought that the need for SUDs treatment increased or remained the same during the most restrictive lockdown periods, they reported that their capacity to meet community demand for SUD services was greatly reduced. This is cause for concern given that prior to the pandemic, access to SUD treatment was already extremely limited in the country, with less than 5% of South Africans able to obtain SUD treatment if required and desired [12]. Our findings suggest that this was partly due to a lack of contingency measures to allow for continued provision of services as well as the lack of COVID 19 guidelines for the provision of substance use services especially at the start of the level 5 lockdown. Similarly, measures to cater for physical distancing protocols and the provision of personal protection equipment (PPEs) for both patients and the treatment workforce were not readily in place at the time of hard lockdown. This reduced capacity to provide treatment should be considered in the light of the country’s bans on the sale and distribution of alcohol and tobacco. A substantial proportion of providers voiced concerns that these bans may have led to feelings of anxiety and distress, particularly among patients who used cigarettes to cope with urges and cravings for other drugs. Restricting access to this coping mechanism at a time of pandemic-related stress [13, 14] may have had the unintended consequence of triggering relapse to substance use.

In addition, study findings suggest that South Africa’s response to the COVID-19 pandemic exacerbated pre-existing structural barriers as well as gender and racial disparities in access to treatment, largely due to worsening socio-economic conditions [15–17]. This finding is in keeping with studies conducted in other contexts that have shown widening disparities in access to SUD treatment – even in countries where telemedicine has been scaled up [18, 19]. Prior to the pandemic, various strategies for reducing financial, geographic access and awareness barriers to SUD treatment had been proposed to address inequities in access SUD services. These included expanding treatment options through integrating SUD treatment into primary care and emergency services [20], changing policy to ensure SUDs are considered a priority health issue requiring appropriate evidence-based treatments including pharmacotherapy [21], and raising SUD treatment literacy among people who use substances, and communities to reduce stigma and facilitate treatment access [22]. Had some of these recommendations been implemented prior to the pandemic, they may have benefitted persons in need of SUD treatment during the pandemic [22]. For instance, accessing SUD treatment in primary health care settings may have reduced both stigma and geographic access barriers associated with obtaining treatment from a stand-alone SUD service [23]. Second, had SUD treatment been recognized as an essential healthcare service, patients would have been permitted to leave their homes to attend SUD treatment, even in the most restrictive periods of lockdown and SUD facilities would have been prioritized for PPE allowing for continued service provision. Third, had individuals with SUD received information on how to safely obtain SUD treatment during the pandemic, this may have alleviated their concerns about engaging with services. With the COVID-19 pandemic exposing the fault lines in South Africa’s SUD services, it is now essential for these recommendations to be implemented—both to address persisting inequities in access to and outcomes of SUD services [24] and to ensure continuity of SUD services in future pandemics [1].

Although some SUD service providers attempted to overcome these challenges to service provision through pivoting from face-to-face patient contact to virtual services via telephone or videoconferencing, this was not without challenges. Providers described low rates of engagement in virtual services largely due to patients having limited access to mobile phones and privacy concerns. Other studies also noted privacy issues and inconsistent access to data, hardware and technology (often due to economic hardship) as barriers to young people (who use substances) engaging in telehealth services in this setting [25]. Arguably these factors may have contributed to the higher rates of disengagement from treatment observed by our participants during this time. This is in contrast to experiences from high-income countries [9, 18, 26, 27], where the use of video call platforms like Zoom and Skype have been viewed as a strategy for ensuring SUD treatment continuity. Nonetheless, emerging evidence from high-income countries highlights growing disparities in access to and continuity of SUD services for economically vulnerable individuals due to difficulties in engaging with telehealth services [28, 29]. In South Africa, where 34.4% of the population are unemployed and the majority live below the poverty line in overcrowded homes and communities [30], tele-SUD treatment is only likely to be a viable mode of treatment delivery for a minority of the population. Further complicating the feasibility of using telehealth to bridge the SUD treatment gap is the fact that most organizations providing SUD treatment are not-for-profit and therefore have limited financial resources to invest in sufficient computer hardware and the technology required to deliver telehealth services. Computers are often outdated and shared among staff. This has been a long-standing issue [31]. Migrating to telehealth would require addressing both patient barriers to participation and organisational resource barriers to the provision of telehealth services.

Findings should be interpreted with caution since the responses obtained in the study were intentionally based around the perspectives of organisations that provide specialist substance use treatment services and not of service users given the urgent need to gain perspectives into service utilisation during the COVID 19 pandemic. We acknowledge that quality of treatment and service users’ experiences of treatment may have changed as a result of the COVID-19 risk mitigation strategies implemented by SUD services. As we did not directly interview service users about how their treatment experience was affected, we were unable to document these perspectives. Given that perceptions of treatment quality are an important predictor of SUD outcomes [32], these perspectives will be important to capture in future research. Furthermore, the survey has limited data on the experiences of people who may have needed specialist substance use care but could not access it during the covid restriction period due to systemic barriers and service availability and therefore our survey is limited to the perspectives of providers only. Whilst, the response rate to the survey was reasonable, access to all service providers may have provided richer data and deeper perspectives in relation to service access and perceived constraints during the COVID 19 restriction period. Finally, the survey results might be subject to bias and not demonstrate a true reflection of specialist substance use services in South Africa, and therefore may not be generalisable.

## Conclusion

Based on the findings it is evident that SUD treatment services in South Africa have been significantly affected during the COVID-19 pandemic and more severely during the onset of the pandemic. Despite ongoing efforts by organizations to put contingency plans in place to help ensure that SUD treatment services would continue to operate during the pandemic, services remain limited and not easily accessible. Our findings suggest that telehealth solutions are not a panacea for South Africa’s persisting inequities in access to SUD treatment but should rather be viewed as one strategy that can be used to enhance access to services. Together with service providers, more effective ways should be sought on how to feasibly expand access to SUD treatment for all South Africans and enhance the country’s preparedness for future health emergencies.

## Data Availability

The datasets used and/or analysed during the current study are available from the corresponding author on reasonable request.
